# Lifestyle and Symptom Management Needs: A Network Analysis of Family Caregiver Needs of Cancer Patients

**DOI:** 10.3389/fpsyt.2021.739776

**Published:** 2021-09-20

**Authors:** Winson Fu Zun Yang, Yiong Huak Chan, Konstadina Griva, Sangita Kuparasundram, Rathi Mahendran

**Affiliations:** ^1^Department of Psychological Science, Texas Tech University, Lubbock, TX, United States; ^2^Department of Psychological Medicine, National University Hospital, Singapore, Singapore; ^3^Biostatistics Unit, Yong Loo Lin School of Medicine, National University of Singapore, Singapore, Singapore; ^4^Lee Kong Chian School of Medicine, Nanyang Technological University, Singapore, Singapore; ^5^Ministry of Health (MOH) Holdings, Singapore, Singapore; ^6^Department of Psychological Medicine, Yong Loo Lin School of Medicine, National University of Singapore, Singapore, Singapore

**Keywords:** caregiver needs, cancer, network analysis, central needs, lifestyle, symptoms management

## Abstract

Previous research on the needs of family cancer caregivers (FCCs) have not elucidated associations between specific caregiving needs. Network analysis, a statistical approach that allows the estimation of complex relationship patterns, helps facilitate the understanding of associations between needs and provides the opportunity to identify and direct interventions at relevant and specific targets. No studies to date, have applied network analysis to FCC populations. The aim of the study is to explore the network structure of FCC needs in a cohort of caregivers in Singapore. FCCs (*N* = 363) were recruited and completed a self-report questionnaire on socio-demographic data, medical data on their loved ones, and the Needs Assessment of Family Caregivers-Cancer scale. The network was estimated using state-of-the-art regularized partial correlation model. The most central needs were having to deal with lifestyle changes and managing care-recipients cancer-related symptoms. The strongest associations were between (1) having enough insurance coverage and understanding/navigating insurance coverage, (2) managing cancer-related pain and managing cancer-related symptoms, (3) being satisfied with relationships and having intimate relationships, and (4) taking care of bills and paying off medical expenses. Lifestyle changes, living with cancer, and symptom management are central to FCCs in Singapore. These areas deserve special attention in the development of caregiver support systems. Our findings highlight the need to improve access to social and medical support to help FCCs in their transition into the caregiving role and handle cancer-related problems.

## Introduction

Family caregivers of cancer patients play a crucial and essential role in care recipients' cancer journey, particularly as patient care moves from inpatient to ambulatory and home settings (1). Throughout this journey, family cancer caregivers (FCCs) themselves may encounter specific needs as a result of many complex factors. A caregiver's need is “unmet” if action or resources taken to attain optimal well-being do not satisfy or resolve the need (2). Previous literature found a consistent association between unmet psychosocial needs and poorer caregiver mental health across the patient's cancer journey (3). Specific unmet needs such as perceived information needs have been associated with higher odds of FCC anxiety (4). While both cancer patients' and FCCs' needs are important as they affect the patient-caregiver relationship (5), quality of life (6), and FCCs' psychological health (7), they may be more dire for FCCs than for patients (2). Therefore, identifying these needs during the cancer patient's treatment journey is crucial in supporting caregiving efforts.

Although research to date has provided much insight on FCCs needs, specific questions on complexities of needs, and their relationships and interactions remain unanswered (8). Needs are complex and increased by interactions, especially at different phases of the disease (9). Recent research methodologies are only able to inform how certain clustered needs in general, such as financial, social, medical, or other needs, influence the caregiving experience. They cannot identify how specific needs in those dimensions affect caregiving or how important specific needs are to FCCs. Identifying specific needs of FCCs can further provide an understanding of the relationship between these needs. For example, FCCs personal time may be restricted as they need to care for their loved ones (10, 11). This may lead to FCCs distancing themselves from family and friends, resulting in an escalation of needs for social and emotional support (2). As such, specific needs may interact and reinforce one another. More importantly, this crucial information may help with effective intervention designs that target these needs or cluster of needs. Therefore, investigating specific needs may answer and provide more insights into the FCC experience.

A novel method to investigate FCC needs and their association is network analysis. Network analysis is an emerging graphical methodology in psychology and has been used to analyze the relationships (edges) between variables (nodes). Complex relationship patterns can be estimated and the network structure can be analyzed to establish core features and properties between nodes (12, 13). Network analysis has been used to investigate associations between symptoms in psychopathology, e.g., symptoms of depression (14) and post-traumatic stress (15). In the context of FCC, it can be used to investigate the relationship between various FCC needs.

The classical theory test or item response theory assumes that constructs arise due to causal interactions between their elements (16). In other words, items do not necessarily arise due to a latent construct, and neither do latent constructs necessarily cause variation in item responses (17). Instead, items are causally dependent on each other to form a network or construct (18). For instance, FCC financial needs do not cause variation in the items; rather, these items covary to cause variation in financial needs. FCCs experience needs due to the accumulation of and interaction between needs in various dimensions and phenomena. For example, FCCs may need to look after their loved ones while sacrificing their own time, learn more about the disease, and manage finances. These distributed and diverse needs come together to form a complex “needs” structure which can inform us on the salient and influencing needs FCCs experience, the relationship among these needs and other needs. Based on this, interventions can be more accurately targeted and introduced to improve FCCs needs, thus modifying and improving the needs structure.

To date, no studies have applied network analysis to examine the needs of FCCs; only two studies have examined symptom experience of cancer patients (19, 20). The aim of this study was therefore (1) to explore the needs of FCCs in Singapore and their interrelatedness via network analysis, and (2) establish the strengths and “centralness” or importance of these needs to FCCs. We hypothesize high interrelatedness of needs, however no a priori hypothesis was made as to which of these were central or of importance to FCCs in Singapore. To our best knowledge, this is the first study to examine FCC needs using network analysis.

## Methods

### Participants and Procedure

FCCs (*N* = 517) of cancer patients (aged 21 and over) followed up in ambulatory clinics at the National University Cancer Institute Singapore were invited to participate in this study. Participants were recruited from May 2017 to December 2017. Inclusion criteria were: (1) Singapore citizens or permanent residents between 21 and 84 years of age, and (2) able to read and understand English. Details on participant recruitment are described elsewhere (21). Convenience sampling method was used as caregivers and their care recipients were most accessible at the clinics. FCCs completed a questionnaire on socio-demography and their care recipient's cancer diagnosis and treatment, and scales to assess their mood state, quality of life, caregiving burden, and needs at home as part of a larger study (21). Four-hundred-and-five participants returned the forms. Forty-two participants were excluded from the analysis for the following reasons: they were not English speakers (*N* = 6), did not complete the NAFC-C (*N* = 25), were not family members (*N* = 9), withdrawal from the study (*N* = 1), and care recipient's diagnosis being revised to “no cancer” (*N* = 1). Hence, a total of 363 participants were included in the final analysis. The study had Ethics Board approval (NUS-IRB Reference No. 2017/000/29, Received: 25 April 2017), and written informed consent was obtained.

### Measures

#### Sociodemographic and Medical Characteristics

Participants completed a self-report questionnaire which collected two types of variables: (1) demographic variables comprising of age, sex, ethnicity, marital status, education, employment, income per capita, and identity of care recipient; and (2) medical variables of the care recipient comprising of type of cancer, cancer stage, and type and length of treatment and whether it was completed.

#### Needs Assessment of Family Caregivers- Cancer (NAFC-C)

The NAFC-C is a 27-item scale that measures different cancer caregiver needs on two dimensions: the importance of the need and the satisfaction with the fulfillment of the need during the past 4 weeks (3). Both dimensions are measured on a five-point Likert-type scale ranging from 0 (Not at all) to 4 (Extremely). Satisfaction rating was reverse coded for each item. For each item, needs score was computed by multiplying satisfaction with importance rating, yielding a range of 0 to 16, with a higher score indicating a higher index of un-fulfillment. The scale consists of four factors (1) psychosocial unmet needs, (2) unmet medical needs, (3) unmet financial needs, and (4) daily activity unmet needs. Our previous study found acceptable validity of the NAFC-C to be used in an Asian population like Singapore (21). In this sample, Cronbach's alpha was strong (α = 0.90).

### Statistical Analysis

Three steps were taken to analyze the data: (1) descriptive statistics, (2) network estimation, and (3) network stability. All analyses were conducted in R 3.5.3 loading on R Studio 1.3.842.

#### Network Estimation

The NAFC-C network was estimated using a Gaussian Graphical Model (GGM), in which edges (associations between needs) represent estimations of partial correlations between nodes (needs). As two nodes are connected in the resulting network, their connections have been controlled for connections to all other nodes in the network. With 27 nodes in the network, there are 351 possible pairwise connections between nodes, and these were estimated in the network model. The least absolute shrinkage and selection operator (LASSO) was applied to the network to identify relevant edges (pairwise connections) and reduce spurious connections, i.e., false-positive connections (22). In short, LASSO shrinks very small edges to zero. The tuning parameter (λ) was selected empirically by applying the Extended Bayesian Information Criterion (EBIC). A more detailed tutorial on how to perform this procedure can be found elsewhere (23).

To examine the importance of each need (node) in the network, strength centrality was computed. A central node exhibits many connections in the network; removing or altering that node will likely result in large changes in the entire network. In short, strength centrality (node strength) measures the relationship between one node and all other nodes in the network. A node with high strength centrality has many connections with other nodes relative to the rest of the network. We only reported strength centrality, as betweenness and closeness centrality were unreliable in recent network analysis (24). Hence, in line with other research we reported node strength only (15). In addition to centrality indices, the predictability of each node will also be calculated using the *mgm* package in R (25). Predictability explains the shared variance of each node with all its direct neighbors (26). It provides an absolute measure of the interconnectedness in the network and, therefore, an idea of the connections' practical relevance (26). In a way, it also quantifies how much a node can be influenced by intervening in all of its neighbors. Higher shared variance between the nodes and their neighbors indicates greater interconnectedness between these nodes.

#### Network Stability

In line with current best practices (27), the accuracy and stability of the network were also estimated using the *bootnet* package (27). To calculate the stability estimates of the centrality indices, each centrality index was bootstrapped 1,000 times with non-parametric samples at 95% confidence interval (27). Centrality stability coefficient ranges from 0 to 1, with values > 0.25 indicate moderate stability and values >0.50 indicate strong stability. Accuracy was investigated by plotting the bootstrapped confidence intervals to examine the variability in the edge weights (27).

#### Identification of Needs of Similar Construct or Processes

In network analysis, some nodes likely measure the same underlying construct, i.e., nodes are collinear. Hence, the *goldbricker* function within the *networktools* package (28) was used to identify potential pairs of nodes that correlate strongly with each other in highly similar patterns with other nodes (topological overlap). In essence, goldbricker identifies pairs of nodes that are strongly inter-correlated (*r* > 0.50) and are sharing at least 75% topological overlap, or <25% of significant divergent dependent correlations at *p* < 0.05 (29).

## Results

### Demographics and Clinical Characteristics

The response rate of the study was 78.33% (*N* = 112 unreturned forms). Table 1 presents the socio-demographic and clinical characteristics of the participants. About three-fifths of the participants were female (*N* = 227, 62.50%), and almost all participants had at least secondary (10 years) education (*N* = 347, 95.60%). The ethnicity distribution for this sample for Chinese (*N* = 263, 72.50%), Malays (*N* = 59, 16.30%), Indians (*N* = 27, 7.44%), and others (*N* = 12, 3.31%) is comparable to the Singapore population ethnicity distribution at 74.35, 13.49, 8.96, 3.21%, respectively (30). Furthermore, cancer distribution among this sample was similar to the latest census in Singapore (31). The most prevalent relationship with care recipients' are parents (*N* = 169, 46.60%), followed by spouses (*N* = 116, 32.00%).

**Table 1:** Participants demographics.

**Socio-demographic and medical variables**	***N* (%^a^)**
**Sex**	
Male	136 (37.50)
Female	227 (62.50)
**Race**	
Chinese	263 (72.50)
Malay	59 (16.30)
Indian	27 (7.44)
Others	12 (3.31)
**Age group (years)**	
21–30	61 (16.80)
31–40	68 (18.70)
41–50	86 (23.70)
51–60	79 (21.80)
61–70	52 (14.30)
71–80	12 (3.31)
**Education**	
No formal education	2 (0.55)
Primary (Some/Completed)	12 (3.31)
Secondary (Some/Completed)/*N*, O Levels / ITE	104 (28.70)
A-Levels/poly diploma	95 (26.20)
Bachelor's degree	116 (32.00)
Masters/Ph.D.	32 (8.82)
**Education (≥High school)**	
Yes	347 (95.60)
No	14 (3.86)
**Marital status**	
Single	119 (32.80)
Married	221 (60.90)
Divorced/Separated	7 (1.93)
Widowed	2 (0.55)
**Employed**	
Yes	238 (65.60)
No	118 (32.50)
**Income (per capita)**	
$2,000 & below	77 (21.20)
$2,001–$8,000	166 (45.70)
$8,001 & above	58 (16.00)
**Relationship with care recipient**	
Spouse	116 (32.00)
Parent	169 (46.60)
Grandparent	8 (2.20)
Son/daughter	19 (5.23)
Sibling	30 (8.26)
Others	1 (3.03)
**Type of cancer**	
Breast	73 (20.10)
Lung	68 (18.70)
Gastro-intestinal/Colorectal/Stomach	59 (16.30)
Hematological/Leukemia/Lymphoma/Myeloma	54 (14.90)
Gynecological	16 (4.41)
Pancreas	11 (3.03)
Multisite	12 (3.31)
NPC/Throat/Oral	13 (3.58)
Renal	8 (2.20)
Brain tumor	6 (1.65)
**Cancer stage if known**	
Early (stages 0–2)	63 (17.40)
Late (stages 3–4)	247 (68.10)
**Is treatment completed?**	
No	265 (73.00)
Yes	83 (22.90)
**Type of treatment completed**	
Chemotherapy	142 (39.10)
Radiotherapy	96 (26.40)
Surgery	147 (40.50)

a *Percentages might not sum up to 100% due to missing data, or rounding difference*.

### Network Estimation

**Figure 1:**
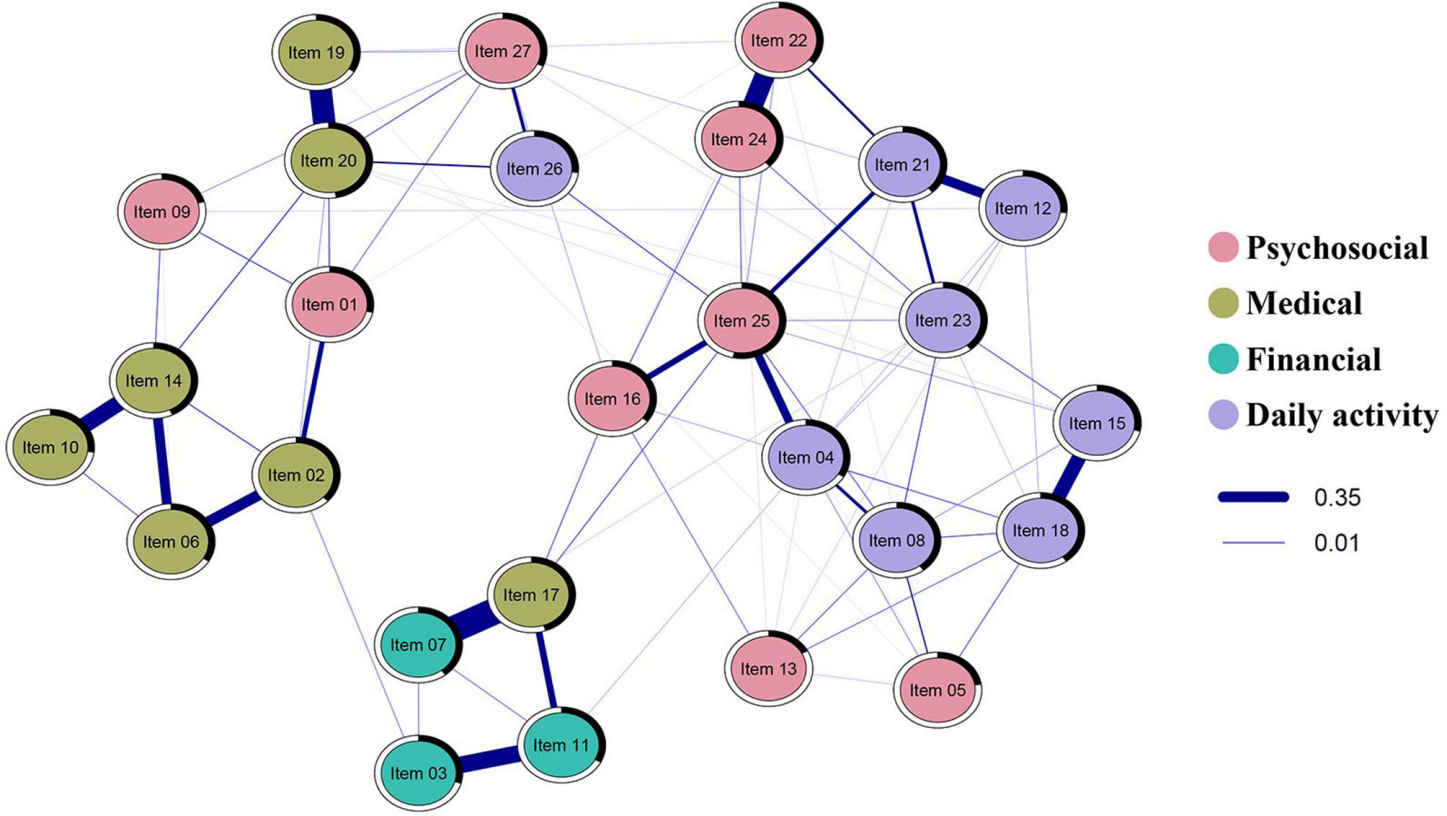
Estimated regularized partial correlation network plot for the NAFC-C (*N* = 363). Only edges that have associations are shown. Blue lines show the positive association between needs; the thickness of the lines represents the strength of the association between needs. The thicker the line, the stronger the relationship between the two needs. The black area around the rings shows the predictability, the variance of a given node explained by all its neighbors. Nodes are colored by their factors in the NAFC; psychosocial needs are colored red, medical needs are colored olive, financial needs are colored green, daily activity needs are colored purple.

**Figure 2:**
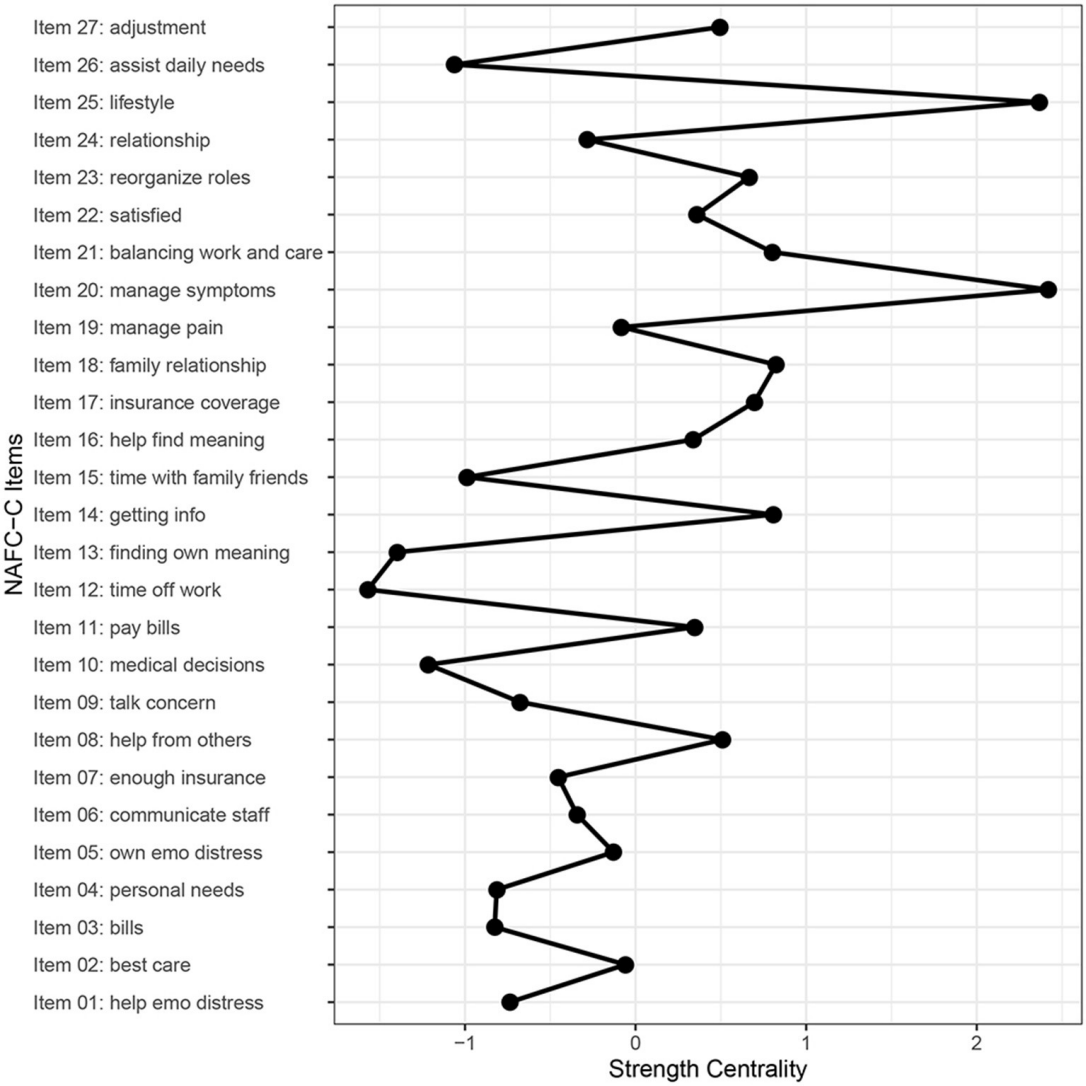
Strength centrality for every item in the NAFC-C. Managing his/her other cancer-related symptoms (item 20) and dealing with lifestyle changes (item 25) had the highest strength while taking time off work (item 12) and finding meaning out of the experience with care recipient's cancer (item 13) had the lowest strength.

Figure 1 shows the estimated network of the NAFC-C, indicating the needs among FCCs. There were 149 non-zero edges out of 351 edges which indicated associations between FCC needs. Furthermore, all needs were positively correlated with each other, with higher values indicating more needs. The strongest edges (depicted by thicker blue lines in Figure 1) emerged between *having enough insurance coverage* (Item 7) and *understanding/navigating insurance coverage* (Item 17), *managing cancer-related pain* (Item 19) and *managing cancer-related symptoms* (Item 20), *being satisfied with relationship* (Item 22) and *having an intimate relationship* (Item 24), and *taking care of bills* (Item 3) and *paying off medical expenses* (Item 11). Of the four pairs, two were associated with financial needs, one was associated with medical needs, and the remaining was associated with psychosocial needs. *Dealing with lifestyle changes* (Item 25) and *managing cancer-related symptoms* (Item 20) had the highest strength centrality (Figure 2).

The average predictability of nodes was 0.34, indicating that, on average, 34% of the variation in one node is explained by its direct neighboring nodes. The predictability of each need is shown in Table 2 and Figure 1 as a black area around the rings. *Dealing with lifestyle changes* (Item 25), *managing cancer-related symptoms* (Item 20), *understanding/navigating insurance coverage* (Item 17), *getting information about cancer diagnosis* (Item 14), *satisfaction with relationship with other family members and friends* (item 18) demonstrated the highest predictability at 0.53, 0.47, 0.45, 0.34, and 0.40, respectively.

**Table 2:** NAFC network metrics.

**Items**	**Description**	**Strength**	**Predictability**
Item 1	Helping his/ her emotional distress (e.g., anger, anxiety, depression, fear, resentment, etc.)	−0.74	0.28
Item 2	Getting the best possible care for him/her	−0.06	0.37
Item 3	Taking care of bills	−0.83	0.29
Item 4	Meeting your personal needs	−0.81	0.34
Item 5	Dealing with your emotional distress (e.g., anger, anxiety, depression, fear, resentment, etc.)	−0.13	0.22
Item 6	Communicating with his/her medical staff	−0.34	0.34
Item 7	Having enough insurance coverage for him/her	−0.45	0.40
Item 8	Getting help from others in order to take time for yourself	0.51	0.40
Item 9	Talking to him/her about his/her concerns	−0.68	0.21
Item 10	Getting involved in medical decisions affecting him/her	−1.21	0.27
Item 11	Paying for his/her medical expenses	0.35	0.32
Item 12	Taking time off work	−1.57	0.27
Item 13	Finding meaning out of your experience with his/her cancer	−1.40	0.17
Item 14	Getting information about the cancer he/she was diagnosed with (e.g., prognosis, treatment, side effects, nutrition)	0.81	0.43
Item 15	Getting together with family and friends	−0.99	0.28
Item 16	Helping him/her find meaning out of cancer	0.34	0.35
Item 17	Understanding/ Navigating medical and/or insurance coverage	0.70	0.45
Item 18	Being satisfied with your relationship with other family members and friends	0.82	0.40
Item 19	Managing his/her cancer-related pain	−0.08	0.34
Item 20	Managing his/her other cancer-related symptoms (e.g., fatigue, nausea)	2.42	0.47
Item 21	Balancing work/school with caring for him/her	0.80	0.38
Item 22	Being satisfied with your relationship with him/her	0.36	0.36
Item 23	Reorganizing roles among family members	0.67	0.39
Item 24	Having an intimate relationship with him/her	−0.28	0.38
Item 25	Dealing with lifestyle changes	2.37	0.53
Item 26	Assisting with his/her daily needs (e.g., preparing meals, transportation, etc.)	−1.06	0.27
Item 27	Helping him/her adjust to life after cancer	0.50	0.32

### Network Stability

The correlation stability coefficient (CS [cor = 0.7] = 0.36) for the strength centrality metric exhibited moderate stability, although it was below the recommended cut-off at 0.50 for strong stability (27). The confidence intervals around the edge weights were large, and most of the confidence intervals overlapped, indicating that their order should be interpreted with caution (see Supplementary Materials for the edge weights).

### Needs Underlying the Same Construct or Process

The identification of needs with a topological overlap revealed six pairs of needs that may have a high conceptual overlap and may be better explained as multiple measurements of the same process or construct: (1) *best possible care* (Item 2) and *communicating with medical staff* (Item 6), (2) *taking care of bills* (Item 3) and *paying for his/her medical expenses* (Item 11), (3) *communicating with medical staff* (Item 6) and *getting information about cancer diagnosis* (Item 14), (4) *getting involved in medical decisions* (Item 10) and *getting information about cancer diagnosis* (Item 14), (5) *managing cancer-related pain* (Item 19) and *managing cancer-related symptoms* (Item 20), and (6) *satisfied with relationship* (Item 22) and *having an intimate relationship* (Item 24). The first five pairs are associated with medical needs, while the last pair is associated with psychosocial needs.

## Discussion

This study aimed to investigate the needs of FCCs via a network approach. We used a partial correlation model applied with LASSO and EBIC to identity the NAFC-C network. Our results revealed that FCCs have critical needs across several distinct domains, i.e., financial needs regarding medical bills and coverage, social and interpersonal relationship changes, and medical-related needs.

Overall, network analysis revealed that connections between needs were positive, confirming our hypothesis. This is not surprising as FCC's needs are highly interrelated (2). However, our analysis revealed that the network has low stability (CS = 0.36), compared to the recommended threshold (CS = 0.50) for strong stability (27). One reason for this is the heterogeneity of the sample. In our study, we had recruited FCCs with care recipients in different phases of the cancer journey (including some who had completed treatment), at different cancer stages, and different cancer types. These factors are associated with distinct needs of care recipients and FCCs. For example, previous research reported that caregiving stress and lack of social support were important needs of FCCs during the early phase of the cancer journey (3). Others however, have shown that although psychological impact persists through the first 6 months of the care recipient's treatment, they reduced over the year and beyond, suggesting that caregivers adapted to their patient's condition over time (32). Similarly, our previous study found that different cancer treatment phases were associated with distinct needs and related outcomes (33). As FCCs adjust to their new roles, their needs evolve largely determined by their care recipient's situation (34). Despite this, the average predictability across all nodes was 0.36, indicating that 36% of the variance of a node that is not predicted by the intercept model is explained by its neighbors. This is an average level of predictability compared to the results of other network analyses (26).

### Core Needs of Caregivers: Lifestyle Changes and Living With Cancer

Network analysis also revealed two important categories of needs: *dealing with lifestyle changes* (Item 25) and managing patients' *cancer-related symptoms* (Item 20). These two needs had the highest strength centrality among other needs, suggesting the important effect and influence of these needs on other needs.

Lifestyle changes are evident during cancer caregiving with FCCs having to adjust and transition from a family member to a caregiver role, sacrifice their personal time and work, and assist their loved ones in daily activities and routines. Our result is in line with previous literature demonstrating that FCCs need to adjust and adopt a new “normal” after sacrificing their jobs, time, space, and life just to care for their sick loved ones (11). This is shown through the strong connection between dealing with lifestyle changes and *balancing work/school with caring for him/her* (Item 21). FCCs face difficulties in trying to relax and manage personal responsibilities due to the additional responsibility of caring for their ill loved ones (10). Furthermore, needs in lifestyle changes were also strongly connected to *meeting personal needs* (Item 4). Our strength centrality results suggest that *helping the loved one find meaning out of cancer* (Item 16) is an important need associated with lifestyle changes. Studies have shown that finding meaning and spirituality in the cancer illness, which can help reduce distress and enhance coping with symptoms, may not be addressed until the disease is in the advanced stages or may even be entirely unaddressed by healthcare teams (35). FCCs themselves may fail to get help in this area and it influences the quality of care they provide and their handling of personal issues (36, 37). Care provision for cancer patients and their caregivers has to be holistic and complementary to psychological, social and medical care to support various aspects of the illness and its progression.

**Figure 3:**
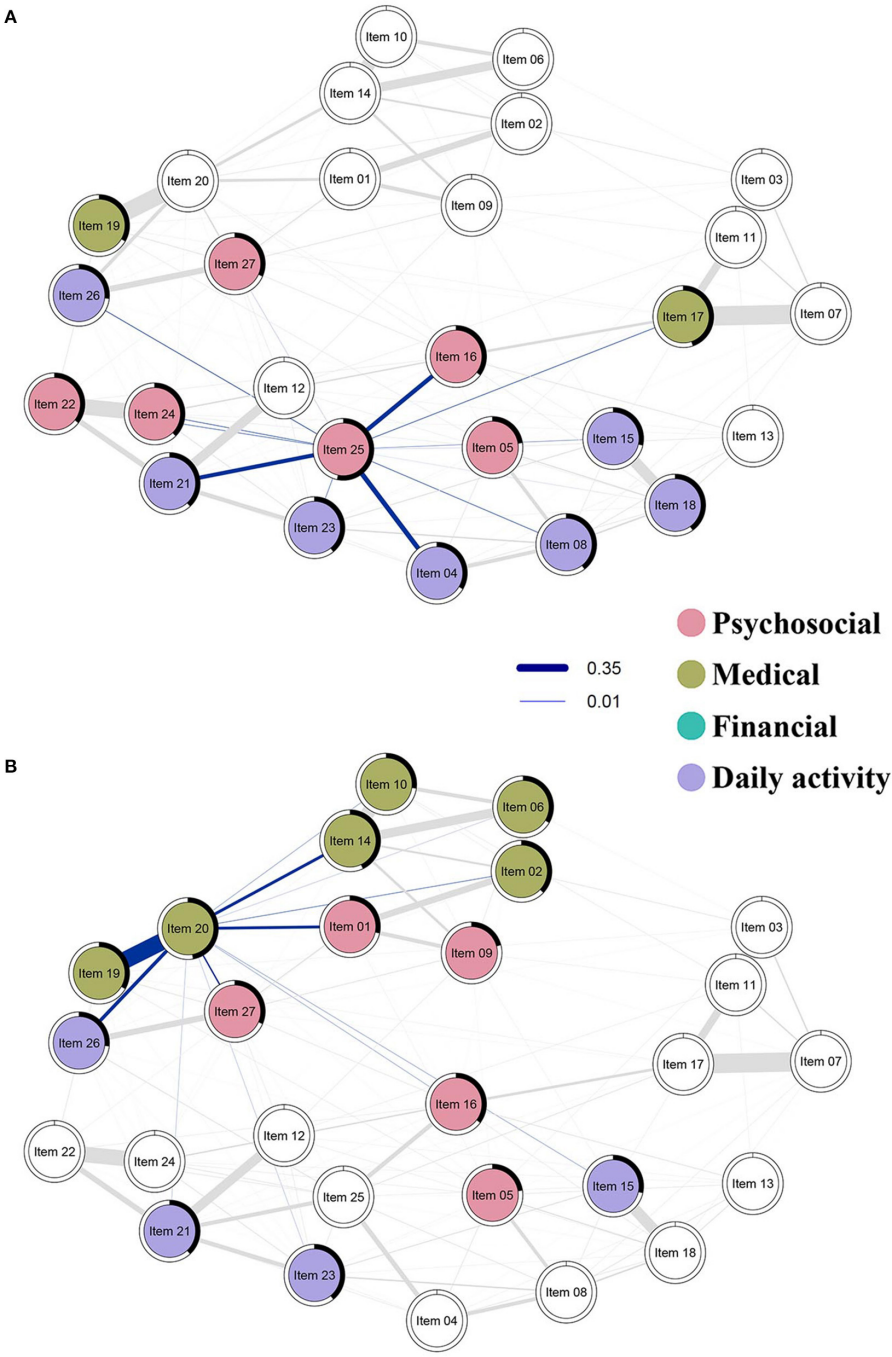
Strength centrality and predictability of top two nodes with highest strength centrality are highlighted in this figure. Blue lines show the positive association between needs; the thickness of the lines represents the strength of the association between needs. The thicker the line, the stronger the relationship between the two needs. The black area around the rings shows the predictability, the variance of a given node explained by all its neighbors. Nodes are colored by their factors in the NAFC; psychosocial needs are colored red, medical needs are colored olive, financial needs are colored green, daily activity needs are colored purple. **(A)** Strength centrality and predictability of dealing with lifestyle changes (Item 25) and **(B)** managing other cancer-related symptoms (Item 20).

Given that FCCs need to find a new stable job if they had previously quit their job or face added responsibilities in their current one, lifestyle changes exacerbate other problems or needs. Our data support this line of reasoning via high predictability in *lifestyle changes* (Item 25) and *satisfaction with the relationship with other family members and friends* (item 18). As predictability quantifies how much a node can be influenced by intervening in all of its neighbors, higher predictability indicates greater interconnectedness between nodes. We found that needs such as *reorganizing roles among family members* (Item 23), *assisting with daily needs* (Item 26), *meeting personal needs* (Item 4), *finding help from others* (Item 8), *and lack of time with family and friends* (Item 15) *are related to satisfaction with the relationship with other family members and friends* (item 18) *and lifestyle changes* (Item 25) (Figure 3A). These factors have become more prominent in recent years as cancer care shifts toward ambulatory care and home settings (1). Eventually, caregivers become lonely and require social support but rarely have the ability nor time to seek help (38). The immense caregiving demands can lead to FCCs to have no time to look after their own personal and social life. This was shown in our data where FCCs needed more time and were unsatisfied with the relationships with their family members and friends, and needed help from others to assist daily needs and other various tasks. Intervening to address these needs will directly impact the personal and social life of FCCs. These needs are therefore critical nodes that policymakers, clinical administrators and service providers need to consider to improve the personal lives of FCCs.

Network analysis also revealed the significance of managing cancer-related symptoms for FCCs. *Managing cancer-related symptoms* (Item 20) had high connections with several other items associated with living with cancer, such as *helping with patient's distress* (Item 1), *getting the best possible care and information* (Items 2 & 14), *managing cancer-related pain and symptoms* (Item 19), and *assisting with life with cancer* (Items 26 & 27) (Figure 3B). This cluster can be seen in two dimensions: managing medical symptoms, and managing psychosocial aspects and daily living, i.e., living with cancer. Caregivers are very involved and invested in the management of medical, psychosocial, and daily needs, of their care recipients. The strong correlation between Items 19 and 20 (cancer related pain and symptoms) demonstrate significant challenges FCCs face in helping their care recipient in dealing with, and managing pain and other symptoms such as fatigue and nausea. Pain is an unpleasant distressing emotional experience with psychological effects, which affect FCCs as well (7, 39). In addition to managing cancer-related symptoms, there were also strong connections for psychosocial distress (Item 1) and assisting with patient needs (Items 26 and 27).

### Providing the Best Possible Medical Care for the Patients

Our analysis also revealed high conceptual overlap between many medical needs, such as *finding the best possible care for the patient* (Item 2), *communicating with medical staff* (Item 6), *getting involved in medical decisions* (Item 10), *getting information about cancer diagnosis* (Item 14) and *managing cancer-related pain and symptoms* (Item 19 & 20). Although there were correlations among items associated with financial needs, they were not of high predictability nor were they central needs of the FCCs. This is not surprising as in Singapore, medical care is comprehensive, readily available and supported by various healthcare financing schemes for its citizens (40). The high topological and conceptual overlap within medical needs suggest that FCCs work diligently to look after their loved ones and provide the best possible care for their care recipient. This result also supports previous studies that demonstrated the involvement of family members in cancer caregiving in Asian societies (41). Duty and filial piety are drivers for FCCs to strive to provide the best possible care for their care recipients, including finding the best medical options for them. Cancer patients and their family often want more and precise communication with their doctors so that they could make the best decisions (42).

### Implications

Our study provides several implications for clinicians and the healthcare administration. Firstly, it must be recognized that while the needs of the FCC and their care recipient can be addressed as a dyad, the FCC and the patient have specific individual needs that need to be addressed separately. While psychosocial support for both FCCs and cancer patients benefits them in coping with daily life, support groups or individualized counseling sessions with advice and interventions based on sociocultural and personal context would provide additional benefit to FCCs. Secondly, healthcare administration must strengthen medical resources, information and awareness on cancers to help patients and FCCs to better manage symptoms and live with cancer; several measures such as psycho-education and case management services can be used to address this. Finally, the strong association with needs for more information, involvement and communication with medical staff suggest the need for streamlined communication channels and easier accessibility, engagement, and regular team meetings with healthcare staff (42). It has been suggested that a concordant model of communication supports all parties in fully participating and sharing perspectives on diagnosis and treatments (43).

### Limitations and Future Research

There are several limitations in this study that need to be addressed. First, this study cannot answer if needs are temporally related; we could only inform their associations. Therefore, we did not speculate if specific needs in one dimension will lead to needs in another dimension. A longitudinal study may be better equipped to answer this important question. Second, our network stability result showed low to moderate stability (CS = 0.36). Upon examining Cronbach's alpha, our data showed very good reliability (Cronbach's α = 0.90, 95% CI = [0.88 0.91]). The NAFC-C was originally developed with the United States population, which has very different population characteristics than the Singapore population (3). This was also shown in our previous validation study as FCCs may interpret items differently from their United States counterpart (21). Hence, other than differences in characteristics of needs, there may be other unexplored needs specific to the Asian population. Given that FCCs have different needs across the patient's cancer journey (34), and we were underpowered to split our sample into different treatment or cancer phases, we were not able to examine the differences and stability of the network structure between different cancer [treatment] phases. This may be better examined with larger sample sizes and in the subgroups. Relatedly, we did not control for possible confounding factors such as demographics and treatment or cancer phases. For example, different ethnic groups in Singapore may have distinct needs which this study could not identify. In the next step, researchers might include these variables to generate a stricter network model or examine differences in needs among ethnicities. Future research using network analysis should also include FCC-related outcomes, especially quality of life, burden, and mood symptoms, to gain insights on how specific needs bridge between other needs and outcomes. Finally, it is now recognized that some cancers can become chronic illnesses similar to other chronic medical conditions. Network analysis might be useful for a more explicit understanding of caregiver needs in the latter group and comparisons of both groups for service planning.

## Conclusions

Overall, our results generate new insights into the needs of FCCs from a network perspective. This study adds relevant and crucial information regarding specific needs for research, social, and clinical support of FCCs, which could not be known through average scores. Needs pertaining to lifestyle changes, living with cancer, and symptom management seem to be central to FCCs in Singapore, and therefore deserve special healthcare administrative attention in developing a support care system for them. FCCs have been found to put effort and time into caring for their care recipients, with less time for themselves. Our findings highlight the need for improved access to and availability of, psychosocial and medical support, to help FCCs with role transitions in caregiving and dealing with cancer illness related problems.

## Data Availability Statement

The data is available from the corresponding author on reasonable request and subject to Ethics Board approval.

## Ethics Statement

The studies involving human participants were reviewed and approved by National Healthcare Group Domain Specific Review Board Ethics Committee. The patients/participants provided their written informed consent to participate in this study.

## Author Contributions

The study was conceptualized by RM, YC, KG, and SK. WY did data analysis with statistical advice from YC. The manuscript was drafted by WY and RM with inputs and reviews by YC, KG, and SK. All authors contributed to the article and approved the submitted version.

## Funding

The research was funded by the Singapore Cancer Society, Cancer Research Grant 2016 (R-177-000-059-592) to RM. The funding body had no involvement in the study design, collection, analysis, or interpretation of data, writing of the manuscript, and the decision to submit the manuscript for publication.

## Conflict of Interest

The authors declare that the research was conducted in the absence of any commercial or financial relationships that could be construed as a potential conflict of interest.

## Publisher's Note

All claims expressed in this article are solely those of the authors and do not necessarily represent those of their affiliated organizations, or those of the publisher, the editors and the reviewers. Any product that may be evaluated in this article, or claim that may be made by its manufacturer, is not guaranteed or endorsed by the publisher.
